# SOX2 plays a crucial role in cell proliferation and lineage segregation during porcine pre‐implantation embryo development

**DOI:** 10.1111/cpr.13097

**Published:** 2021-07-11

**Authors:** Mingyun Lee, Kwang‐Hwan Choi, Jong‐Nam Oh, Seung‐Hun Kim, Dong‐Kyung Lee, Gyung Cheol Choe, Jinsol Jeong, Chang‐Kyu Lee

**Affiliations:** ^1^ Department of Agricultural Biotechnology Animal Biotechnology Major, and Research Institute of Agriculture and Life Sciences Seoul National University Gwanak‐gu Korea; ^2^ Research and Development Center Space F corporation Hwasung Korea; ^3^ Institute of Green Bio Science and Technology Seoul National University Pyeongchang Korea

**Keywords:** CRISPR/Cas9, SOX2, pig, embryo

## Abstract

**Objectives:**

Gene regulation in early embryos has been widely studied for a long time because lineage segregation gives rise to the formation of a pluripotent cell population, known as the inner cell mass (ICM), during pre‐implantation embryo development. The extraordinarily longer pre‐implantation embryo development in pigs leads to the distinct features of the pluripotency network compared with mice and humans. For these reasons, a comparative study using pre‐implantation pig embryos would provide new insights into the mammalian pluripotency network and help to understand differences in the roles and networks of genes in pre‐implantation embryos between species.

**Materials and methods:**

To analyse the functions of SOX2 in lineage segregation and cell proliferation, loss‐ and gain‐of‐function studies were conducted in pig embryos using an overexpression vector and the CRISPR/Cas9 system. Then, we analysed the morphological features and examined the effect on the expression of downstream genes through immunocytochemistry and quantitative real‐time PCR.

**Results:**

Our results showed that among the core pluripotent factors, only SOX2 was specifically expressed in the ICM. In SOX2‐disrupted blastocysts, the expression of the ICM‐related genes, but not OCT4, was suppressed, and the total cell number was also decreased. Likewise, according to real‐time PCR analysis, pluripotency‐related genes, excluding *OCT4*, and proliferation‐related genes were decreased in *SOX2*‐targeted blastocysts. In SOX2‐overexpressing embryos, the total blastocyst cell number was greatly increased but the ICM/TE ratio decreased.

**Conclusions:**

Taken together, our results demonstrated that SOX2 is essential for ICM formation and cell proliferation in porcine early‐stage embryogenesis.

## Graphical abstract

We analysed the phenotypes of SOX2 knockout and SOX2‐overexpressing pre‐implantation porcine embryos to understand the function of SOX2, one of the core transcription factors. In SOX2‐targeted embryos, total cell number decreased, and expression of pluripotent‐related genes and proliferation‐related genes decreased. Loss‐ and gain‐of‐function analysis revealed that SOX2 regulates total cell number and expression of pluripotent‐related genes and proliferation‐related genes. These results indicate that SOX2 plays important role in cell proliferation and ICM formation in porcine pre‐implantation embryos.

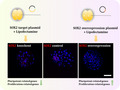

## 1 INTRODUCTION

In mammals, two lineage segregations occur during pre‐implantation embryo development. First, the cell population is divided into the inner cell mass (ICM) and trophectoderm (TE), and second, the ICM is divided into the epiblast (EPI) and primitive endoderm (PrE).^1^ ICM cells are pluripotent and eventually differentiate into foetal and extraembryonic tissues. In contrast, TE cells are involved in implantation and give rise to the placenta.^2^ Much effort has been focused on uncovering the important factors and mechanisms of lineage specification. Initially, the reciprocal inhibition of the target genes of OCT4 and CDX2 was found in mouse embryonic stem cells (ESCs), and this mechanism was expected to determine the first lineage specification.^3^ However, advances in the single blastomere transcriptomics and embryo cell tracking system combined with transgenic techniques present a different theory.^4^, ^5^ Recent studies have demonstrated that the biased formation of CARM1 and paraspeckles at the 4‐cell stage in mouse embryos leads to the differential expression of ICM‐specific transcription factors such as SOX2 and SOX21.^5^, ^6^, ^7^ In addition, studies that reveal signalling pathways and mechanisms related to lineage specification are active in pig pre‐implantation embryos.^8^, ^9^, ^10^, ^11^ These studies can be a basis for post‐implantation development studies by identifying the exact cell development pathway in the embryonic stage. Furthermore, it can provide new insights for the study of naïve and primed pluripotent states and the differentiation pathways in pluripotent stem cells.

Mammalian embryo development has species‐specific differences in the pre‐implantation developmental process, ICM‐specific gene expression patterns, and ESC pluripotent signalling. Although the initial developmental process is similar between species, there is a difference in the timing of differentiation into hypoblasts and epiblasts. The second lineage specification occurs between days 3.5 and 4.5 of development in mouse ICM, day 6 of development in humans and days 6 to 7 of development in pigs.^12^ Additionally, ICM‐specific gene expression patterns expressed during embryo development differ from species to species. SSEA1 is detected in the ICM of mouse and pig embryos,^13^ whereas SSEA3, SSEA4, TRA‐1‐60, TRA‐1‐81 and GCTM‐2 were expressed in ICM of human blastocysts.^14^ Furthermore, OCT4 expression in mouse blastocysts is limited to the ICM, whereas OCT4 in porcine and human blastocysts is expressed in both the ICM and TE.^15^, ^16^, ^17^ In addition, the signalling required for maintaining the pluripotency of mouse ESCs differs from species to species. While leukaemia inhibitory factor and bone morphogenetic protein 4 signalling play crucial roles in maintaining the pluripotency of mouse ESCs,^18^ in humans, ESC pluripotency is maintained through extracellular signal‐regulated kinase and ACTIVIN/NODAL signalling pathways.^19^ Recent studies have shown that activation of the non‐canonical WNT pathway in porcine ESCs is important for maintaining pluripotency.^20^ Since these pluripotency‐related mechanisms differ from species to species, species‐specific studies are required to elucidate pluripotency networks. In particular, OCT4, SOX2 and NANOG are core transcription factors that lead to pluripotency, which should be the starting point of the species‐specific pluripotency analysis.^21^, ^22^

Transgenic embryos were first produced in 1974 by injecting the SV40 virus into early‐stage mouse embryos. Later, advances in molecular biology and reproductive physiology established several methods for producing transgenic embryos. In particular, endonuclease‐mediated genetic engineering has developed greatly, and the first report of a zinc finger nuclease (ZFN) knockout mammalian embryo model was in 2009.^23^ Similar to ZFN, transcription activator‐like effector nucleases (TALENs) that rely on the Fok1 endonuclease were first used to generate genetically modified embryos in 2011.^24^ Although ZFN and TALEN have been widely used to generate genetically modified embryos, they have now been replaced by clustered regularly interspaced short palindromic repeat (CRISPR) and CRISPR associated (Cas9) because of their simplicity and flexibility in targeting.^25^ Recently, there have been a number of comparative studies in the mammalian embryo field,^26^ which have used the CRISPR/Cas9 technology to accelerate research.^27^, ^28^ However, since only a limited number of species have been studied, we tried to determine the pluripotent network in the pig embryo, which is recognized as a model animal.^29^, ^30^, ^31^ Therefore in this study, we investigated the expression patterns of core transcription factors and the role of SOX2 in parthenogenetically activated (PA) porcine embryos. First, we investigated gene expression patterns at each stage during early embryogenesis to select an ICM‐faithful marker. Then, we injected CRISPR/Cas9 vectors into a 2‐cell stage embryo to knock out the *SOX2* gene. After the culture period, the transcript and protein expression patterns of the control blastocyst and *SOX2*‐targeted blastocyst were analysed by immunostaining and qPCR in the early and late blastocyst stages. Next, we induced overexpression by microinjecting exogenous *SOX2* into the embryos, and morphological differences and gene expression patterns were evaluated at the blastocyst stage. This approach revealed the role of SOX2 in the development of pre‐implantation embryos and will aid in understanding the mechanism of lineage segregation.

## 2 MATERIALS AND METHODS

The care and experimental use of pigs were approved by the Institute of Laboratory Animal Resources, Seoul National University (SNU‐140328‐2). Unless otherwise stated, all chemicals were obtained from Sigma‐Aldrich Corp.

### 2.1 In vitro embryo production

The ovaries of the prepubertal gilts were obtained from a local slaughterhouse (Anyang‐si, Gyeonggi‐do) and transferred to the laboratory in warm saline. Cumulus‐oocyte complexes (COCs) were collected by aspirating 3‐7 mm follicles of the prepubertal gilts using a 10 mL syringe with an 18‐gauge needle. Sediments were washed with TL‐HEPES‐PVA medium, and oocytes with compact cumulus cells and granulated cytoplasm were selected for in vitro maturation. The washed COCs were cultured in tissue culture medium (TCM‐199; Life Technologies) containing 10 ng/mL epidermal growth factor, 1 mg/mL insulin and 10% porcine follicular fluid for 44 hours at 39°C at 5% CO_2_ and 100% humidity. The COCs were matured with 10 IU/mL gonadotropin hormone, pregnant mare serum gonadotropin (Lee Biosolutions) and human chorionic gonadotropin for the first 22 hours. The COCs were then matured under hormone‐free conditions. To generate parthenotes, cumulus‐free oocytes were activated with an electric pulse (1.0 kV/cm for 60 ms) in activation medium (280 mmol L^−^ mannitol, 0.01 m mol L^−^ CaCl_2_ and 0.05 m mol L^−^ MgCl_2_) using a BTX Electrocell Manipulator (BTX), followed by 4 hour of incubation in PZM3 medium containing 2 mmol/L 6‐dimethylaminopurine.

### 2.2 Production of CRISPR/Cas9 vectors and SOX2 gene expression vector

The candidate targeting sequence for against the pig *SOX2* gene was selected using the CRISPR gRNA design tool (https://chopchop.cbu.uib.no) to improve gene‐targeting efficiency and minimize off‐targeting effects. DNA oligonucleotides carrying the target sequences were constructed by adding PAM sequences (Table S1). The candidate DNA construct for *SOX2* was inserted into the pX330 plasmid and validated using the pCAG‐EGxxFP reporter system.^32^ The SOX2‐pX330 constructs and pCAG‐EG(pig SOX2)FP constructs were introduced into porcine foetal fibroblast (pFF) cells plated in 6‐well plates (300 ng/well) using the Lipofectamine 3000 Reagent (Thermo Fisher Scientific). EGFP fluorescence was observed under a fluorescence microscope at 48 hours after transfection. Genotyping of genomic DNA of the transfected pFF was extracted using the G‐spin Total DNA Extraction Kit (iNtRON Biotechnology). Genomic DNA samples were amplified using 10 pmol of porcine SOX2‐specific primers (Table S1) and 2 ×PCR master mix solution (iNtRON Biotechnology). Amplified PCR products were subjected TA cloning and analysed by an ABI PRISM 3730 DNA Analyzer (Applied Biosystems). Finally, the selected guide sequence was inserted into the pX458 vectors for microinjection into embryos.

The porcine *SOX2* coding sequence was synthesized and replaced in the human SOX2 site of the pCXLE‐hS + EGFP vector (Addgene #74945). For plasmid construction, Gibson Assembly using the NEB Builder Hifi DNA Assembly (New England Biolbas) was used. All the primers used for cloning are listed in Table S1. All vectors were verified by nucleotide sequencing.

### 2.3 Cytoplasmic injection of the DNA‐lipofectamine complex

For the microinjection assay, 10 μL of 90 ng/μL DNA in combination with 1 μL of Lipofectamine^33^ (Stem reagent; Thermo Fisher Scientific) was incubated for 5 min in Media‐199 (Gibco), and the final DNA concentration was 15 ng/μL. One day after PA, the embryos at the 2‐cell stage were injected with two pl of plasmid‐lipofectamine solution in manipulation media. The microinjection procedure was conducted using a micromanipulator (Eclipse TE2000, Nikon) with a Femtotip Ⅱ microinjector (Eppendorf). After microinjection, the embryos were washed and then cultured in PZM3 media for 5 days.

### 2.4 Immunocytochemistry

Each stage of embryos without zona pellucida was fixed in 4% paraformaldehyde for 15 minutes at room temperature. The fixed samples were permeabilized using 1% Triton ×‐100 for 1 hours at room temperature and then washed three times with phosphate‐buffered saline (PBS). The embryos were blocked using 10% goat serum or donkey serum in PBS for 1 hours at room temperature. Samples were stained with anti‐SOX2 (5 μg/mL), anti‐NANOG (1 μg/mL), anti‐OCT4 (1 μg/mL) and anti‐SOX17 (1 μg/mL) antibodies in PBS containing 10% goat serum or donkey serum at 4°C overnight (Table S2). After washing three times in washing solution (PBS with 0.2% Tween‐20 and 1% BSA for 10 minutes), the embryos were incubated with goat anti‐rabbit Alexa 594 (Invitrogen) or donkey anti‐rabbit Alexa594 (Invitrogen) antibodies in PBS with 10% goat serum or donkey serum at RT for 1 hours. All samples were washed three times with washing solution after secondary antibody treatment. Immunostained embryos were mounted on a slide glass with Prolong Gold with DAPI (Invitrogen) and cured for more than 24 hours. We have described the list of antibodies in Table S2. Images of stained cells were captured using an inverted fluorescence microscope and processed by the ImageJ program.

### 2.5 Quantitative real‐time polymerase chain reaction

Total RNA from pooled embryos at each stage of in vitro–produced embryos (2‐3 cells, n = 20; 4 cells, n = 20; 6‐8 cells, n = 20; morula, n = 10 and blastocysts, n = 5) was isolated using an Arcturus^®^ PicoPure^®^ RNA Isolation Kit (Applied Biosystems) following the manufacturer's instructions. cDNA was synthesized using a High‐Capacity RNA‐to‐cDNA Kit (Applied Biosystems). The cDNA samples were amplified using Power SYBR Green Master Mix (Applied Biosystems) containing 1 pmol of each primer set listed in Table S3 in a 10 μL reaction volume. Amplification and detection were conducted using the ABI 7300 Real‐Time PCR System (Applied Biosystems) under the following conditions: one cycle of 50°C for 2 minutes and 95°C for 10 minutes, followed by 40 cycles of denaturation at 95°C for 15 seconds and annealing/extension for 1 minutes (annealing/extension temperatures were dependent on each primer set). The dissociation curves were analysed, and the amplified products were loaded onto gels to confirm the specificity of the PCR products. The relative expression level was calculated by normalizing the threshold cycle (Ct) values of each gene to that of the reference gene beta‐actin (ACTB) via the delta‐delta Ct method.

### 2.6 Statistical analysis

Statistical analysis of data was performed using GraphPad Prism Software (version 7). Significant differences in gene expression among the experimental groups were determined by one‐way analysis of variance followed by Tukey's multiple‐comparison test. Total cell number analysis of the impact of SOX2 overexpression was assessed by Chi‐square analysis. Differences were considered significant at *P* < .05 (*P < .05 and **P < .01 in the figures). Data are presented as the mean ± standard error of the mean (SEM).

## 3 RESULTS

### 3.1 Expression analysis of core pluripotency transcription factors in porcine pre‐implantation embryos

**FIGURE 1:**
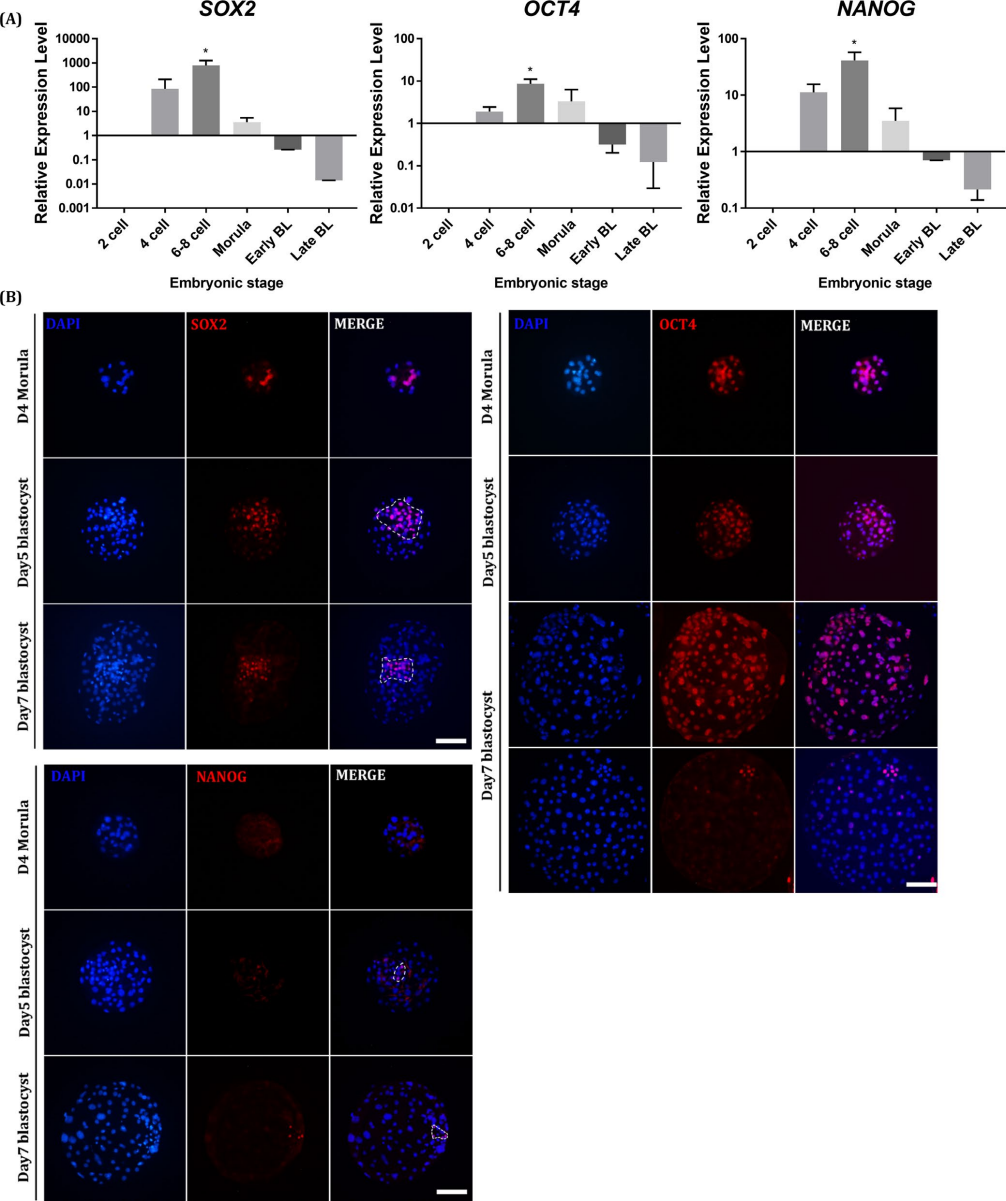
Core pluripotency factor expression pattern in preimplantation porcine embryos A, Expression levels of core pluripotency genes(OCT4A, SOX2, NANOG) were measured in 6 developmental stages (2 cells, 4 cells, 6‐8 cells, early blastocyst and late blastocyst). Error bars represent the mean SEM, * indicates a significant difference between groups. B, Expression and localization of core pluripotency genes in 3 developmental stages (morula, early Blastocyst, late Blastocyst) of embryos. Nuclei were stained with DAPI, and SOX2, OCT4A, and NANOG were stained in red. The size marker corresponds to 100 μm

In mammals, numerous genes are involved in lineage segregation during pre‐implantation embryo development, of which *OCT4*, *SOX2* and *NANOG* are reportedly crucial for establishing and maintaining the pluripotent cell population known as the ICM and epiblasts.^34^, ^35^, ^36^, ^37^, ^38^, ^39^ Pigs have an extraordinarily longer pre‐implantation embryo development than mice and humans.^40^ The distinct features of the pre‐implantation stages lead to differences in the pluripotency network in pig embryos. To identify lineage‐specific markers, the spatiotemporal pattern of the gene expression of *OCT4*, *SOX2* and *NANOG* was analysed in porcine pre‐implantation embryos derived from parthenogenesis. The expression of all pluripotency genes in the pooled embryos was upregulated in the 6‐ to 8‐cell stage and then decreased starting from the morula stage, as determined by qPCR (Figure 1A). Next, to assess the spatial gene expression pattern, the expression of OCT4, SOX2 and NANOG was examined in porcine morula and 5‐day‐old (D5) (early) and 7‐day‐old (D7) (late) blastocysts through immunocytochemistry (ICC) assays (Figure 1B). Whereas OCT4 is an ICM‐specific marker in mouse pre‐implantation embryos,^41^ our data showed that pig OCT4 was highly expressed in both the TE and ICM from the morula to the D7 blastocyst stage. More specifically, in several blastocysts, OCT4 was exclusively detected in the ICM population, indicating that OCT4 expression was gradually restricted to ICMs starting from the late blastocyst stage in pigs. NANOG, known as a marker gene of epiblasts, was not observed in the morula stage but was starting to be expressed in a subset of cells from the D5 blastocyst stage. Interestingly, SOX2 was partially expressed in a subset of blastomeres at the morula stage and, in turn, was exclusively observed in the ICM of blastocysts, as shown in a previous study.^17^ These results show that SOX2 plays a more crucial role than OCT4 and NANOG in the first lineage segregation, in which ICM/TE lineage specification occurs, in porcine pre‐implantation embryos. Therefore, we studied the role of SOX2 during porcine pre‐implantation embryo development through its gain and loss of function.

### 3.2 Effects of *SOX2*‐targeting plasmid microinjection on embryo development

**FIGURE 2:**
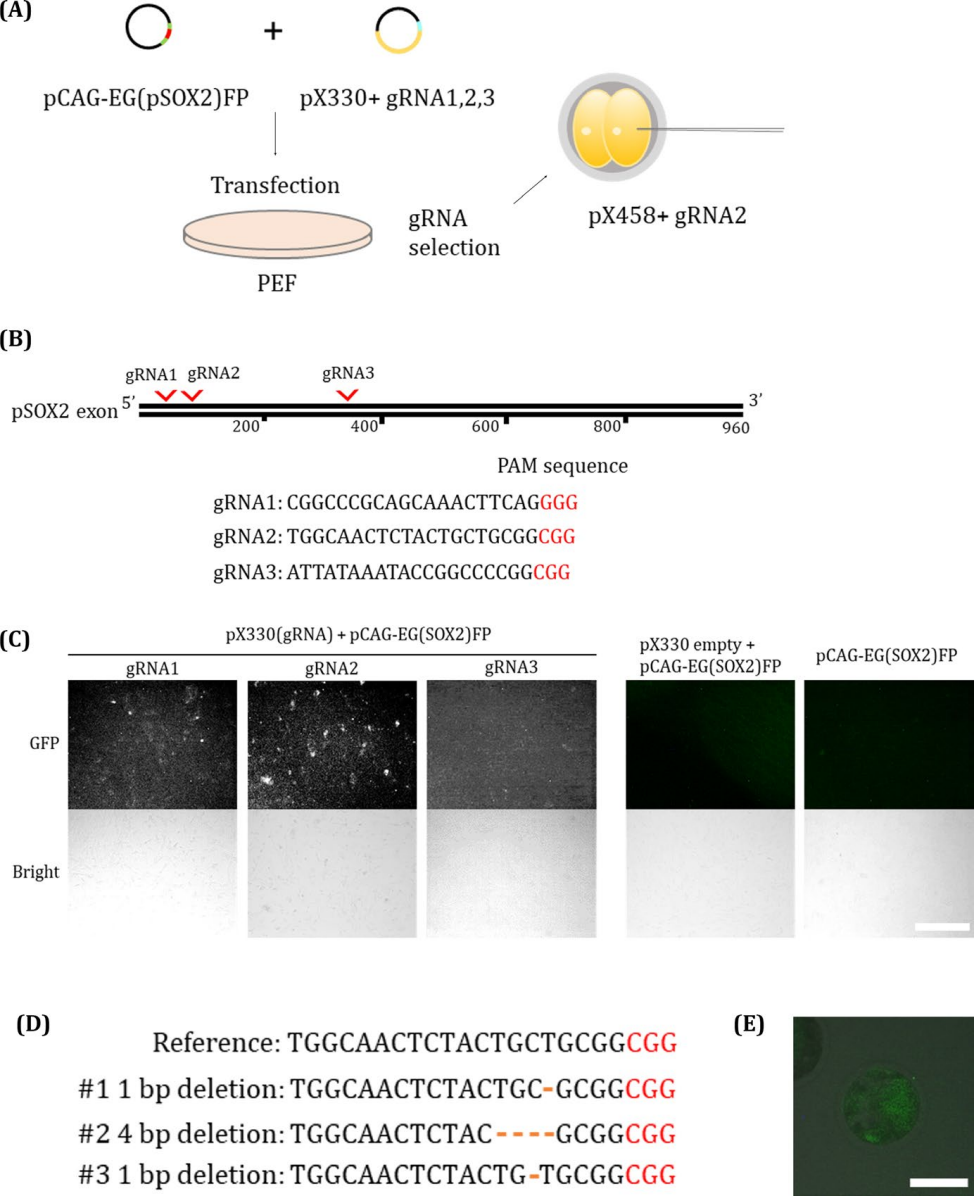
Experimental scheme and CRISPR gRNA validation. A, Experimental scheme of SOX2 targeting of porcine embryos. B, Porcine SOX2 locus and gRNA targeting sites. PAM sequences are indicated in red font. C, The cleavage efficiency of pX330, which contained the gRNA 1 to 3 sequences, in the porcine SOX2 region of the pCAG‐EGXXFP vector. D, Deletion mutations in porcine embryonic fibroblasts transfected with pX330 (gRNA2). E, Early blastocyst microinjected with pX458 (gRNA2) Lipofectamine complex

**FIGURE 3:**
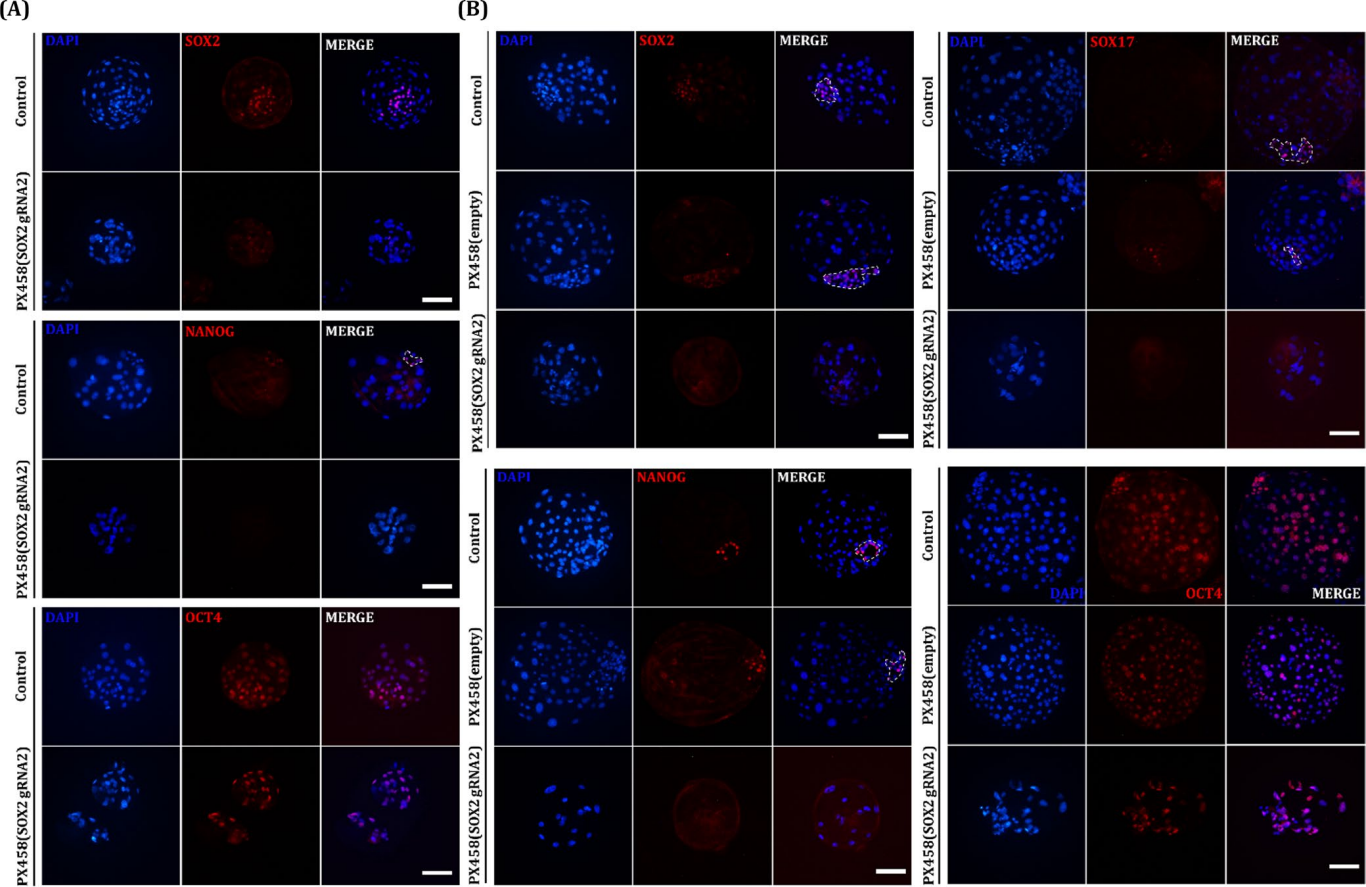
Targeting of SOX2 prevents expression of ICM specific gene in porcine embryos Immunofluorescence analysis for pluripotent genes (SOX2, NANOG, OCT4 ‐red) and DAPI nuclear staining blue in uninjected and pX458 + gRNA injected porcine early BL. Sample sizes of each groups were n = 10. Immunofluorescence analysis for Pluripotent genes (SOX2, NANOG, OCT4 ‐red) and DAPI nuclear staining blue) in uninjected, pX458 injected, pX458 + gRNA injected porcine late BL Sample sizes of each groups were n = 10. Size marker corresponds to 100 µm

To edit the endogenous *SOX2* locus, we selected and cloned three targeting sequences for pig SOX2 (Figure 2B) into the pX330 vector according to a previously reported protocol.^42^ The gRNA cleavage efficiency was evaluated by the pCAG‐EGxxFP validation system (Figure 2A). The negative control did not express GFP, and the gRNA2‐targeted group showed the highest expression of GFP among the three gRNA‐treated groups (Figure 2C). Furthermore, the frameshifts occurred in target site by inducing indels with gRNA2, as determined by genomic DNA sequencing (Figure 2D). Based on this observation, we use gRNA2 to downregulate *SOX2* in pig embryos. To investigate whether SOX2 plays an important role in ICM formation in pre‐implantation porcine embryos, pX458‐gRNA2 containing Cas9 and EGFP was injected into embryos using a micromanipulator. Because knockout of *SOX2* immediately followed by parthenogenetic activation caused a negative effect on the developmental efficiency of embryos in a preliminary study (data not shown), microinjection was performed at the 2‐cell stage embryos (Figure 2A). EGFP expression was maintained until the blastocyst stage, which confirmed that the plasmid vector was efficiently introduced and worked in pig embryos by microinjection (Figure 2E). Immunofluorescence analysis revealed that SOX2 protein expression was downregulated in the pX458‐gRNA2‐injected embryos at D5 blastocyst stage (Figure 3A). SOX2‐positive cells were significantly decreased in pX458‐gRNA‐2‐injected blastocysts compared to the control group (Table 1). Similarly, the number of NANOG‐positive cells was reduced by *SOX2‐*knockout blastocysts. Likewise, in the D7 blastocyst stage, SOX2‐ and NANOG‐positive cells and cells positive for SOX17, a primitive endoderm marker, were also significantly decreased (Figure 3B, Table 1). Furthermore, the total cell number of blastocysts decreased in D7 blastocysts injected with pX458‐gRNA2 (Table 1). However, OCT4 was still stably expressed in most of the blastocyst cells of blastocyst even after *SOX2* knockout, suggesting that OCT4 and SOX2 are regulated by an independent pluripotency network in pigs.

**TABLE 1:** The number of total cells and pluripotent marker positive cells

Group	No. blastocysts (n = 3)	Cells in blastocysts			
		Total cell number	SOX2‐positive cells	NANOG‐positive cells	SOX17‐ positive cells
Cont.	30	131.8 ± 8.4^a^	17.6 ± 1.6^a^	6.8 ± 1.4^a^	7.2 ± 1.6^a^
PX458 empty	30	117 ± 6.5^a^	13.4 ± 1.6^a^	4.7 ± 0.8^a^	6.2 ± 1.0^a^
PX458‐SOX2 gRNA	30	29 ± 2.5^b^	1.6 ± 0.6^b^	0.8 ± 0.4^b^	0.3 ± 0.2^b^

Note

The number of cells was counted in the late blastocyst. Values with different letters (a and b) are significantly different (*P* < .05).

**FIGURE 4:**
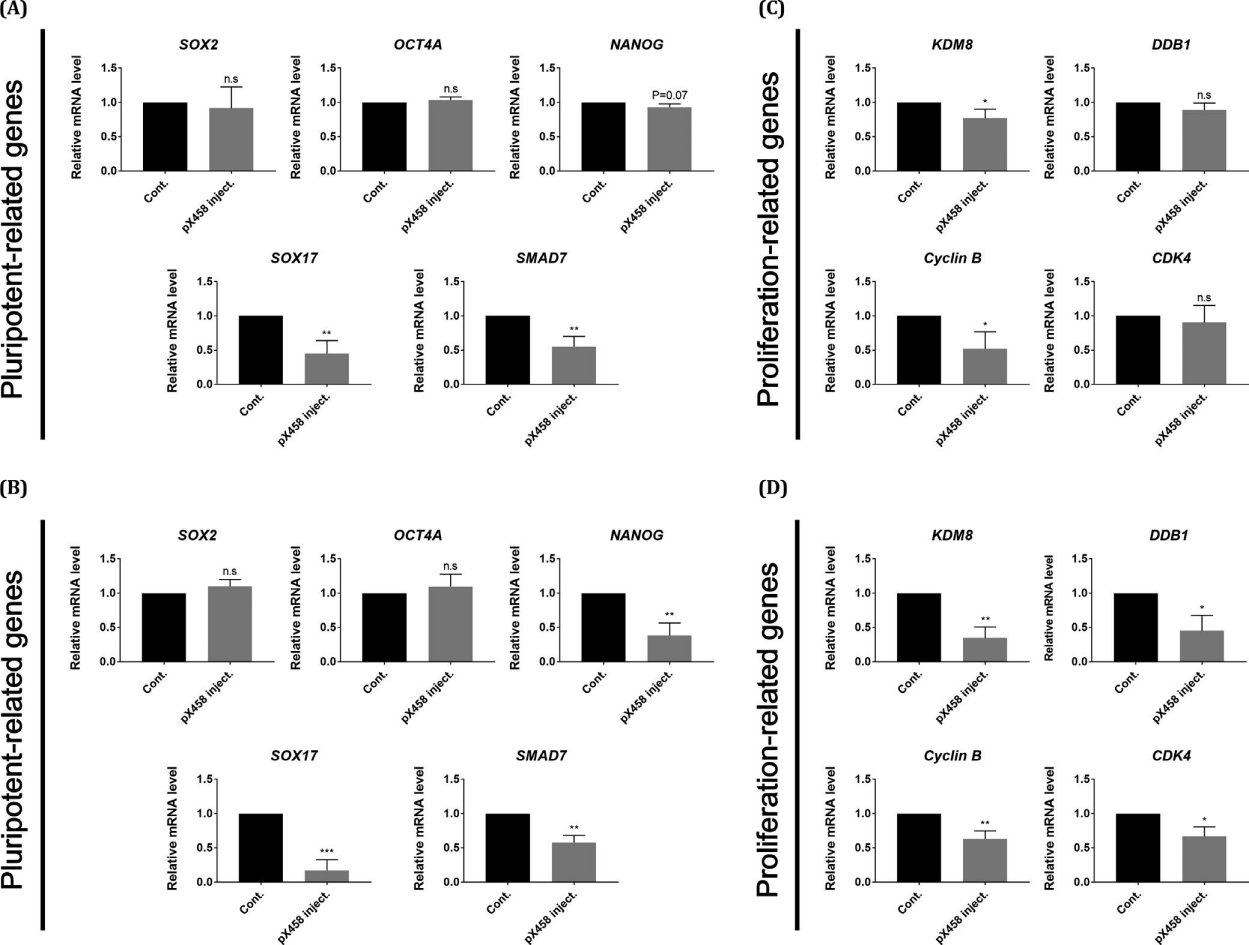
Gene expression patterns of proliferation and pluripotency genes in SOX2‐targeted porcine embryos A, B Transcription levels of pluripotency‐related genes shown for control SOX2‐targeted day 5 blastocysts and day 7 blastocysts. C, D Transcription levels of proliferation‐related genes shown for control SOX2‐targeted day 5 blastocysts and day 7 blastocysts. Each group has three replicates. *corresponds to significant differences. (*: *P* < .05, **: *P* < .01, ***: *P* < .001)

qPCR analysis showed that while the expression levels of *SOX2*, *OCT4* and *NANOG* were not significantly different between the control and SOX2‐knockout groups at the D5 blastocyst stage, as determined by qPCR (Figure 4A), the expression of *NANOG* was significantly reduced at the D7 blastocyst stage (Figure 4B). *SOX17*, a primitive endoderm marker gene, and *SMAD7*, a regulator involved in ESC self‐renewal, were downregulated in SOX2‐knockout blastocysts at both the D5 and D7 blastocyst stages.^43^ In the case of proliferation‐related genes, *KDM8*, which is known to regulate embryonic cell proliferation as a direct target of SOX2 and *Cyclin B*, a regulator of cell mitosis was downregulated when SOX2 was knocked out in embryos (Figure 4C,D).^44^, ^45^ *DDB1*, known to play an important role in embryo proliferation,^46^ and *CDK4*, a key protein for G1‐to‐S phase transition,^47^ were significantly decreased in the *SOX2*‐knockout embryos at the late blastocyst stage. These findings suggest that targeting SOX2 in porcine embryos reduces the expression of ICM‐specific genes except OCT4 and has a negative effect on cell proliferation. Taken together, these data suggested that SOX2 regulates the ICM formation and cell proliferation of porcine pre‐implantation embryos in an OCT4‐independent manner.

### 3.3 Overexpression of exogenous *SOX2* through microinjection assay

**FIGURE 5:**
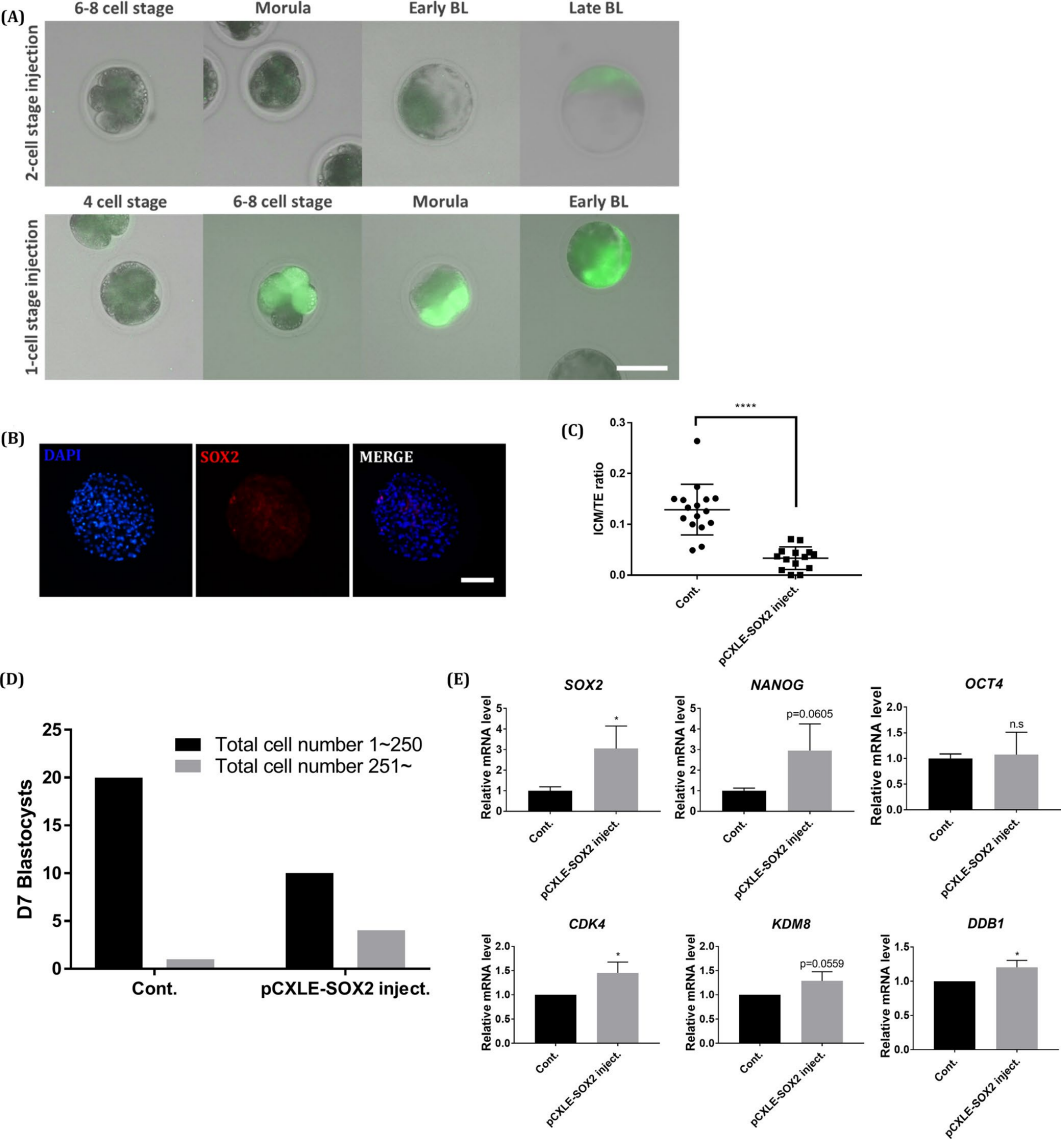
Analysis of SOX2‐overexpressing porcine blastocysts. A, GFP expression during the development of embryos injected with pCXLE‐pSOX2 at the 1‐cell stage and 2‐cell stage. The size marker corresponds to 100 μm. B, Immunofluorescence analysis for SOX2 (red) and DAPI nuclear staining in SOX2‐overexpressing day 7 blastocysts. The size marker corresponds to 100 μm. C, Cell allocation to the ICM and TE in control and SOX2‐overexpressing blastocysts. D, Number of blastocysts per total cell number in control and SOX2‐overexpressing D7 blastocysts. E, Transcription levels of pluripotency‐related genes and proliferation‐related genes in control and SOX2‐overexpressing D7 blastocysts

Finally, to better define the function of SOX2 during pig pre‐implantation embryo development, SOX2‐overexpressing embryos were analysed. To induce *SOX2* overexpression during embryogenesis, microinjection of pCXLE‐pSOX2 into blastomeres was implemented in 1‐ and 2‐cell stage embryos. The embryo injected into the 1‐cell stage expressed EGFP overall, and the embryos injected into the 2‐cell stage expressed a mosaic pattern. Most of the 1‐cell stage SOX2‐overexpressing embryos were arrested at the morula stage or D5 blastocyst stage and did not form a normal blastocoel (Figure 5A), while CMV‐GFP plasmid‐injected embryos and media (TCM‐199)‐injected embryos developed to the blastocyst stage (Table 2). However, the 2‐cell stage injected embryos could develop into D7 blastocysts and hatch from the zona pellucida. These results indicate that SOX2 overexpression hampers the lineage specification during blastocyst formation, and subsequent analysis was conducted by plasmid injection in 2‐cell stage embryos.

**TABLE 2:** Developmental rates of SOX2‐overexpressing embryos

Group	No. embryos (n = 3)	No. cleaved (%)	Blastocyst (%)
pCXLE‐pSOX2 1C stage injected	101	71 (71.7 ± 0.03)	8 (8.1 ± 0.03)
CMV‐GFP 1C stage injected	91	71 (77.9 ± 0.05)	32 (35.2 ± 0.01)
Media 1C stage injected	90	68 (75.6 ± 0.03)	26 (28.9 ± 2.94)

As revealed by immunostaining analysis, the ICM/TE ratio decreased in the SOX2‐overexpressing blastocyst group (Figure 5B, C). In contrast, blastocysts with over 250 cells produced more blastocysts in the *SOX2*‐overexpressing group than in the control group (Figure 5B, D). In the qPCR assay, the expression of *SOX2* and *NANOG* increased compared to the control, but *OCT4* showed no significant difference (Figure 5E). Furthermore, the expression levels of *CDK4*, *KDM8* and *DDB1* associated with proliferation were increased. These results indicate that SOX2 plays important roles in cell proliferation and ICM formation in porcine pre‐implantation embryos.

## 4 DISCUSSION

### 4.1 *SOX2* plays an important role in the first lineage specification of pig pre‐implantation embryos

SOX2, along with OCT4 and NANOG, is one of the core transcription factors and is specifically expressed in the ICM in mammalian embryos.^17^, ^48^, ^49^ In addition, SOX2 is also considered a key transcription factor for pluripotency because it is expressed in ESCs and germ cells.^21^, ^50^ For these reasons, SOX2 is used to reprogram somatic cells into induced pluripotent stem (iPS) cells, or else incomplete reprogramming occurs.^51^, ^52^ In mice, OCT4 is a key factor that drives ICM formation via the downregulation of CDX2 during the first lineage segregation.^53^ OCT4 and CDX2 are exclusively expressed in the ICM and TE, respectively, in mouse blastocysts. However, in pigs, it has been reported that OCT4 is expressed in both the ICM and TE and is co‐expressed with the TE marker CDX2 from the D5 blastocyst stage.^54^ Furthermore, since downregulation of OCT4 prevents TE formation, OCT4 expression is essential for TE lineage specification.^55^ It seems that a longer period of pre‐implantation embryo development in ungulates, including pigs compared to rodents and primates, leads to different molecular mechanisms during embryogenesis.^12^ A previous study shows that instead of OCT4, SOX2 is an authentic marker of ICM in porcine pre‐implantation embryos,^17^ which revealed that SOX2 could be the key gene during the first lineage specification of pig pre‐implantation embryos.

In this study, we modulated the expression of SOX2 to determine its roles during pig embryo development. Consistent with previous results, *SOX2*‐knockout porcine embryos formed blastocoels but failed to form ICMs, which confirmed that SOX2 is involved in ICM formation. Additionally, 2‐cell stage asymmetric *SOX2*‐overexpressing embryos could develop into blastocysts while most embryos overexpressing SOX2 at the 1‐cell stage were arrested at the morula stage, which indicates that asymmetric distribution of SOX2 before the morula stage is essential for lineage segregation. Recent studies revealed that Sox2 is required to derive an ICM population along with OCT4 in mouse embryo development.^7^, ^56^ Mouse embryos with disruption of SOX2 achieved blastocyst formation but failed to complete lineage segregation.^38^ Taken together, these results suggest that SOX2 plays an important role in first lineage specification via asymmetric distribution before the morula stage during porcine pre‐implantation embryo development. Additionally, as revealed in previous studies, different species‐specific molecular mechanisms for the pluripotency network and lineage specification exist between mouse and pig embryo development.^57^

Interestingly, unlike *NANOG*, the mRNA expression of *OCT4* and the protein expression of OCT4 were not influenced by overexpression or knockout of *SOX2* in porcine embryos. In previous studies, it was shown that *Carm1‐* and *LincGET*‐overexpressing mouse embryos show a significant change in the expression levels of *SOX2* and *NANOG* but not *OCT4*.^7^ Moreover, as a result of quantifying the binding of OCT4 and SOX2 to genomic DNA in mouse embryos, it was shown that SOX2 interacts with DNA in a long‐lived manner while OCT4 interacts in a short‐lived manner at the 4‐cell stage, which indicates that SOX2 is regulated independently from OCT4.^56^ In another study, although NANOG is downregulated in human ESCs followed by OCT4 knockout, SOX2 expression is stably maintained.^58^ Correspondingly, in OCT4‐knockout bovine embryos, the expression of NANOG is suppressed, but the first lineage segregation is not affected.^36^ Accordingly, SOX2 regulates the ICM formation of porcine pre‐implantation embryos in an OCT4‐independent manner although they are both crucially involved in ICM formation.

### 4.2 Mitogenic effects of *SOX2* on pig pre‐implantation embryos

Various pluripotency‐related transcription factors reportedly can affect cellular proliferation as well. Changes in cell cycle dynamics are related to the pluripotency state, and differentiation can be regulated through regulation of the proliferation regulator.^59^ Moreover, since the proliferation rate of embryos is an important factor in determining embryo quality,^60^ it is necessary to investigate the interrelationship with pluripotency genes. OCT4 and SOX2 affect the cell cycle,^61^, ^62^ and downregulation of OCT4 caused a significant decrease in the total cell number in human embryos.^58^

Our data suggest that SOX2 is required for embryo proliferation (Table 1, Figure 5B). These results match those mentioned in studies of SOX2‐targeted embryos in mice.^38^ Likewise, previous studies showed that the overexpression of SOX2 enhances proliferation in human mesenchymal stem cells and Wharton's jelly stem cells.^62^, ^63^ Additionally, SOX2 is strictly required for proliferation in human MSCs and primordial germ cells.^47^, ^50^ In the above studies, overexpression of SOX2 triggers high expression of Cyclin D1, accelerating the G1‐to‐S transition. Based on these findings, we speculate that SOX2 promotes cell proliferation during embryo development as well as in pluripotent cells and multipotent stem cells.

### 4.3 Gene manipulation techniques to define the pluripotency network using mammalian embryos

Transgenesis in mammalian embryo studies has contributed to gaining deeper insight into the pluripotency network during embryo development. The CRISPR‐Cas9 system, which has been studied extensively in recent years, has improved efficiency and is being used for genetic modification research. CRISPR‐Cas9 has been used to clarify the role of genes during pre‐implantation development. A recent study revealed that OCT4/OCT4 has different functions in human and mouse embryogenesis.^58^ In bovine embryos, it was found that OCT4 is required for NANOG expression by *OCT4* disruption.^36^ NANOG is essential for epiblast formation and maintenance of pluripotency using *NANOG*‐targeted bovine embryos.^39^ Alternatively, lineage specification in mammalian embryos is being studied through overexpression analysis. The roles of OCT4 and NANOG in embryos were analysed by somatic cell nuclear transfer (SCNT) of stably overexpressed cell lines to embryos.^64^, ^65^ Recently, RNA direct cytoplasmic injection was performed for overexpression to control embryonic cell fate.^7^, ^54^

There are differences in the gene expression systems: the RNA‐based system induces immediate expression, whereas the DNA‐lipofectamine system is delayed by ZGA (zygotic genome activation). ZGA starts at the 4‐cell stage in pigs, and previous research has shown that gene expression occurs later in bovine embryos than murine embryos with microinjection of DNA plasmids.^66^, ^67^ As the expression of exogenous *SOX2* was delayed by ZGA, it did not affect the early stage of embryonic gene expression. Therefore, SOX2‐overexpressing embryos may appear to generally develop into blastocysts. Thus, the DNA‐lipofectamine system can be used as a method by delaying the timing of gene knockout in embryo research. The gene expression system can be used differently depending on the species, and the timing of exogenous gene expression can be controlled.

## 5 CONCLUSION

In conclusion, in the present study, we attempted to assess the role of SOX2 during pig embryo development by gene editing technology. SOX2, as an authentic marker of ICM in porcine pre‐implantation embryos, plays an important role in the first lineage specification via asymmetric distribution before the morula stage during porcine pre‐implantation embryo development. Additionally, it regulates the ICM formation and cell proliferation of porcine pre‐implantation embryos in an OCT4‐independent manner although both these genes are crucially involved in ICM formation. However, it remains to be elucidated how different species‐specific molecular mechanisms regulate the pluripotency network during mammalian embryo development. Finally, our data provide new insights into the mammalian pluripotency network and help to understand the differences in the roles and networks of genes in pre‐implantation embryos between species.

## CONFLICTS OF INTEREST

The authors declare no conflicts of interest.

## AUTHOR CONTRIBUTIONS

M. Lee, J.‐N. Oh, S.‐H. Kim, K.‐H. Choi, D.‐K. Lee and C.‐K. Lee designed research; M. Lee, J.‐N. Oh, S.‐H. Kim, GC. Choe and J. Jeong performed research; M. Lee, J.‐N. Oh, S.‐H. Kim, K.‐H. Choi and D.‐K. Lee analysed data; M. Lee and C.‐K. Lee wrote the paper and C.‐K. Lee finally approved the manuscript.

## ETHICAL APPROVAL

The authors assert that all procedures in this work complied with the ethical standards of the relevant national and institutional guides on the care and use of laboratory animals. The Institutional Animal Care and Use Committee, Seoul National University, approved the care and experimental use of pigs (SNU181024‐8).

## DATA AVAILABILITY STATEMENT

The data sets in this study are available from the corresponding author on reasonable request.

## Supporting information

Table S1‐3
