# Supplementary material for: SOX2 plays a crucial role in cell proliferation and lineage segregation during porcine pre‐implantation embryo development
Source: Cell Prolif. 2021 Jul 11;54(8):e13097. doi: 10.1111/cpr.13097 (PMC8349655; doi:10.1111/cpr.13097)
Supplement: Supplementary file 1 — Table S1‐3 [file CPR-54-e13097-s001.docx]

**Table S1 Primers used in this study.**

| Primers | Sequence (5’→3’) | Description |
| --- | --- | --- |
| pCAG-pSOX2 | F: tgaggatccgctagcctgcaATGTACAACATGATGGAGAC | pCAG-EG(porcine SOX2)FP construction |
|  | R: atcgaattcgtcgacctgcaGCCGGCGCCCACCCCGACCC |  |
| pCXLE- pSOX2 | F: gtctcatcattttggcaaagCCCGCATGTACAACATGATG | pCXLE-porcine SOX2 construction |
|  | R tgactagtccccgaagcttgGGCCCTCACATGTGAGAGAG |  |
| SOX2 gRNA-1 | F: CACCGCGGCCCGCAGCAAACTTCAG | gRNA-1 on-target site |
|  | R: CGCCGGGCGTCGTTTGAAGTCCAAA |  |
| SOX2 gRNA-2 | F: CACCGTGGCAACTCTACTGCTGCGG | gRNA-2 on-target site |
|  | R: CACCGTTGAGATGACGACGCCCAAA |  |
| SOX2 gRNA-3 | F: CACCGATTATAAATACCGGCCCCGG | gRNA-3 on-target site |
|  | R: CTAATATTTATGGCCGGGGCCAAAC |  |
| Porcine SOX2 | F: ATGTACAACATGATGGAGAC | *SOX2* specific primers |
|  | R: TCGGGACCACACCATGAAAG |  |

Underlined nucleotides refer to gene-specific regions, and lowercase letters indicate overhangs.

**Table S2 List of antibodies.**

| Primary Antibodies | Target | Host | Company | Catalog Number |
| --- | --- | --- | --- | --- |
|  | SOX2 | Rabbit | Millipore | AB5603 |
|  | OCT4 | Rabbit | Santa Cruz | sc-9081 |
|  | NANOG | Rabbit | Peprotech | 500-P236 |
|  | SOX17 | Goat | R&D systems | AF1924 |
| Secondary Antibodies | Fluorescent dye | Target/Host | Company | Catalog Number |
|  | Alexa594 | Rabbit/Goat | Invitrogen | A-11012 |
|  | Alexa594 | Rabbit/Donkey | Invitrogen | A-21207 |

**Table S3 Oligonucleotide sequences used in quantitative PCR.**

| Primer | Sequence (5’→3’) | Tm | Size |
| --- | --- | --- | --- |
| OCT4 | F: GCTGGAGCCGAACCCCGAGG | 68°C | 150 |
|  | R: CACCTTCCCAAAGAGAACCCCCAAA |  |  |
| SOX2 | F: CGGCGGTGGCAACTCTAC ' | 64°C | 100 |
|  | R: TCGGGACCACACCATGAAAG |  |  |
| NANOG | F: CATCTGCTGAGACCCTCGAC | 60°C | 195 |
|  | R: GGGTCTGCGAGAACACAGTT - |  |  |
| SOX17 | F: GCAAGATGCTGGGCAAGT | 60°C | 112 |
|  | R: TTGTAGTTGGGGTGGTCCTG |  |  |
| KDM8 | F: CACGGATGAGGAGTGGTCCCAG | 60°C | 142 |
|  | R: GCTGATGTCCTGCTTCAGCTCC |  |  |
| DDB1 | F: CATTCCTCGTTCCATCCTGATG | 60°C | 128 |
|  | R: CCTTCTTACGGTCGCTCAACAG |  |  |
| SMAD7 | F: TGTCCAGATGCTGTGCCTTCCT | 60°C | 143 |
|  | R: CTCGTCTTCTCCTCCCAGTATG |  |  |
| CYCLIN B | F: TGGCTAGTGCAGGTTCAG | 60°C | 199 |
|  | R: CAGTCACAAAGGCAAAGT |  |  |
| CDK4 | F: 5′-GCATCCCAATGTTGTCCG | 60°C | 126 |
|  | R: 5′-GGGGTGCCTTGTCCAGATA |  |  |
| ACTB | F: GTGGACATCAGGAAGGACCTCTA | 60°C | 131 |
|  | R: ATGATCTTGATCTTCATGGTGCT |  |  |
